# A novel method of microsatellite genotyping-by-sequencing using individual combinatorial barcoding

**DOI:** 10.1098/rsos.150565

**Published:** 2016-01-20

**Authors:** Salla Vartia, José L. Villanueva-Cañas, John Finarelli, Edward D. Farrell, Patrick C. Collins, Graham M. Hughes, Jeanette E. L. Carlsson, David T. Gauthier, Philip McGinnity, Thomas F. Cross, Richard D. FitzGerald, Luca Mirimin, Fiona Crispie, Paul D. Cotter, Jens Carlsson

**Affiliations:** 1 Area 52 Research Group, University College Dublin, Belfield, Dublin, Republic of Ireland; 2 School of Biology and Environment Science, University College Dublin, Belfield, Dublin, Republic of Ireland; 3 Earth Institute, University College Dublin, Belfield, Dublin, Republic of Ireland; 4 Carna Research Station, Ryan Institute, National University of Ireland, Galway, Carna, Connemara, Republic of Ireland; 5 Evolutionary Genomics Group, Research Programme on Biomedical Informatics (GRIB), Hospital del Mar Research Institute (IMIM), Universitat Pompeu Fabra (UPF), Barcelona 08003, Spain; 6 School of Biological Sciences, Queen’s University Belfast, Medical Biology Centre, Lisburn Road, Belfast, UK; 7 Department of Biological Sciences, Old Dominion University, Norfolk, VA, USA; 8 Beaufort Fish Genetics Programme, School of Biological, Earth and Environmental Sciences/Aquaculture and Fisheries Development Centre, University College Cork, Distillery Fields, North Mall, Cork, Republic of Ireland; 9 Marine and Freshwater Research Centre, Galway-Mayo Institute of Technology, Dublin Road, Galway, Republic of Ireland; 10 Teagasc Food Research Centre, Moorepark, Fermoy, Cork, Republic of Ireland; 11 Alimentary Pharmabiotic Centre, Cork, Republic of Ireland

**Keywords:** amplicon sequencing, *Gadus morhua*, genotyping by sequencing, next-generation sequencing, ssr, universal primer

## Abstract

This study examines the potential of next-generation sequencing based ‘genotyping-by-sequencing’ (GBS) of microsatellite loci for rapid and cost-effective genotyping in large-scale population genetic studies. The recovery of individual genotypes from large sequence pools was achieved by PCR-incorporated combinatorial barcoding using universal primers. Three experimental conditions were employed to explore the possibility of using this approach with existing and novel multiplex marker panels and weighted amplicon mixture. The GBS approach was validated against microsatellite data generated by capillary electrophoresis. GBS allows access to the underlying nucleotide sequences that can reveal homoplasy, even in large datasets and facilitates cross laboratory transfer. GBS of microsatellites, using individual combinatorial barcoding, is potentially faster and cheaper than current microsatellite approaches and offers better and more data.

## 1. Introduction

The advent of next-generation sequencing (NGS) technologies has fundamentally changed how genetic sequence data are generated [1]. While NGS was primarily introduced to substantially increase sequence yield for genome projects [2–4], it has in addition enabled high-throughput genotyping that can be used for genetic studies, including population genetics, by using a range of protocols (e.g. RAD [5], ddRAD [6], 2bRAD [7]). This is collectively known as ‘genotyping-by-sequencing’ (GBS) [8,9].

The primary advantage of GBS for population genetic studies is the generation of increased quantities of data that allows for improved statistical power and high genome representation [9]. The concurrent development of single-nucleotide polymorphism (SNP) genotyping platforms (e.g. SNP-chips [10,11], microfluidic TaqMAN assays [12]) and the persistent problems associated with microsatellite genotyping has led to a shift from using microsatellites to SNPs as the preferred marker for genetic studies [13]. The main problems with capillary and gel-based microsatellite studies include fragment size homoplasy, poor levels of inter-laboratory calibration, the genotype not including the underlying sequence information and inherently laborious genotyping [14,15]. However, there are also unresolved problems regarding SNPs, such as ascertainment bias, transferability among SNP genotyping platforms and the requirement for high template DNA quality [16–18]. While SNPs do not suffer many of the issues associated with microsatellite genotyping, the major advantage of microsatellites over SNP-based approaches for population analyses is the higher statistical power per locus [19–21]. Additionally, microsatellites are preferred to SNPs in forensic, parentage and kinship studies owing to their higher mutation rates and polyallelic nature [22–25].

Many of the issues associated with microsatellite-based population studies could be mitigated using a GBS approach. The effects of size-homoplasy [26] can be effectively eliminated because the genotype incorporates the underlying sequence information. The difficulties of inter-laboratory calibration would be significantly reduced, as GBS considers actual base-pair lengths of the alleles, and not the estimated allele size sequence length [15]. Additionally, the elimination of time consuming capillary or gel electrophoresis runs [27] can reduce genotyping time, thereby increasing efficiency. Further, GBS has the potential to use established microsatellite multiplex panels, enabling calibration with existing datasets and facilitating inter-laboratory collaboration. However, few GBS studies based on microsatellites have been published [28–30].

The current study aims to assess the potential of microsatellite GBS using Atlantic cod *Gadus morhua* (L.) as the study organism. The primary objective was to examine the potential of microsatellite GBS using *de novo* and existing capillary/gel electrophoresis-based multiplex marker panels. The secondary objective was to develop a rapid and cost-effective method for microsatellite GBS, that can take advantage of modern NGS platforms, using combinatorial barcoding for implementation in large-scale population genetics studies.

## 2. Material and methods

A total of 64 Atlantic cod were collected from the Celtic Sea (*n*=32) and the West of Ireland (*n*=32) in 2011. Tissue samples were preserved in molecular grade ethanol. DNA was extracted using a Chelex^®^ protocol [31]. DNA was quantified using a NanoDrop 1000 Spectrophotometer (Thermo Scientific, Wilmington, DE, USA) and diluted to a concentration of 50 ng μ*l*^−1^. These samples had previously been genotyped in six original PCR multiplexes at 53 microsatellite loci using a conventional ABI capillary-based approach [32] thus providing a platform for direct comparison with the current study.

Six multiplex PCR panels comprising 53 microsatellite loci [32] were analysed to test the capability of microsatellite GBS on all individuals. Each of the six original multiplex panels included loci in three different size classes (class I: 115–213 bp, class II: 203–320 bp and class III: 265–416 bp, see the electronic supplementary material, table S1). To assess the preferential amplification of shorter fragments during PCR and NGS sequencing [30,33], weighted ratio mixtures of fluorescently labelled amplicons were visualized on an ABI 3130xl Genetic Analyzer (Applied Biosystems; conditions according to [32] suggesting an optimal size-class amplicon-ratio of 1(I) : 1(II) : 8(III).

Three PCR size-class-combinations for GBS were evaluated (electronic supplementary material, table S1). The first *de novo* multiplex panels were weighted for amplicon size by combining all markers of the same size classes (3-PCR) from the six original multiplexes using a subset of 16 individuals from the Celtic Sea. The second size-class-combination consisted of the existing multiplexes (6-PCR) using the same subset of 16 individuals from the Celtic Sea (*sensu*[32]). The third PCR size-class-combination comprised the six existing multiplex panels, subdivided into three smaller multiplex panels according to the three size classes using all 64 individuals (18-PCR).

**Figure 1.**
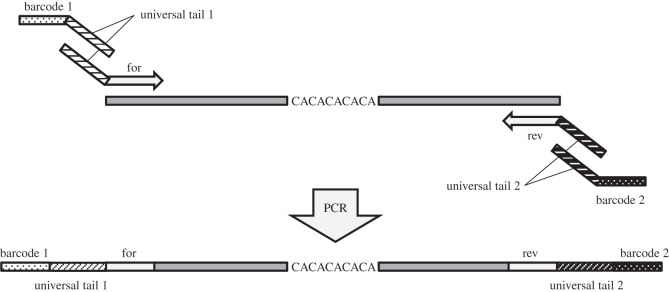
Diagram of the four-primer PCR and the structure of the resulting amplicon.

The three-primer PCR approach [32,34] was modified into a four-primer PCR to produce unique individual combinatorial barcoded amplicons suitable for pooled amplicon NGS in population genetics studies. Each barcode comprised 10 bp of synthesized DNA sequence [35]. The modified protocol consisted of locus-specific primers (forward and reverse) that were adapted to include universal primer sequence (figure 1; electronic supplementary material, table S2). Two barcoded universal primers were included to incorporate two barcodes into each of the resulting amplicons. In total, 12 forward and eight reverse DNA barcodes allowed for the recovery of 96 unique individual combinatorial barcodes (electronic supplementary material, table S3).

Multiplex PCRs (cycling condition as in [32]) were performed in 10 μl reactions (size classes I and II) and 20 μl reactions (size class III, in 3-PCR and 18-PCR size-class-combinations) with 1 μl template DNA, 1× Multiplex PCR Master Mix (Qiagen, Hilden, Germany), 2 pmol of each locus-specific primer and 8 pmol of each barcoded universal primer per locus-specific primer with the corresponding universal primer sequence. For each multiplex and PCR size-class-combination equal amounts of PCR product from each individual were pooled and separated by gel electrophoresis on a 2% agarose gel at 5 V cm^−1^. PCR products were visualized using Safe Imager^®^ (Invitrogen, Life Technologies, Carlsbad, CA, USA). Products corresponding to expected amplicon sizes were cut out from the gel and placed into 2 ml centrifuge tubes. DNA was extracted using the QIAquick^®^ Gel Extraction Kit (Qiagen, Hilden, Germany). Amplicon DNA was quantified using Qubit^®^ 2.0 Fluorometer with a dsDNA HS Assay kit (Life Technologies). Amplicons from the 3-PCR and 18-PCR size-class-combinations were normalized to the optimal size-class amplicons-ratio (1 : 1 : 8). The amplicons were concentrated using the Amicon^®^ Ultra 30 K procedure (Merck Millipore Ltd., Merck KGaA, Darmstadt, Germany). An aliquot of the final library was diluted to a concentration of 500 ng in 16 μl for sequencing on a 454 Roche^®^ platform ([36]; 454 Life Sciences Corp., a Roche Company, Branford, CT, USA).

Roche adaptors were added to the amplicon library using the Rapid Library Preparation Kit [37]. Amplicon concentrations were estimated using quantitative PCR to assess differential amplification success depending on amplicon size. Amplicons were sequenced on two 1/4 regions of a Pico TiterPlate using a 454 GS-FLX Sequencer with titanium reagents at Teagasc Food Research Centre, Moorepark, Ireland. The Shotgun Data Processing Pipeline was used for signal processing to increase sequence yield [38].

A python script was developed to process raw sequence data by identifying sequence reads containing the forward and reverse (combinatorial) barcodes and the locus-specific primers and grouping them accordingly. The script was based on the Levenshtein distance metric which measures the distance between two sequences of characters. The error rate of Roche 454 GS-FLX amplicon sequences when including both sequencing and PCR errors has been estimated in *G. morhua* at 6% [39]. It was therefore necessary to allow for sequencing and PCR errors in the reads in order to avoid a significant loss of reads during the identification and grouping process. The python script allowed for up to two and three sequencing errors (both substitutions and indels) in the combinatorial barcodes and primers, respectively, to reflect a potential sequencing and PCR error rate of approximately 6%. All the scripts used are available on github (https://github.com/egenomics/micomba).

Grouped and classified sequences were imported into Geneious^®^ 7 as fasta files and organized into folders per locus per individual. Loci were manually genotyped by viewing all of the reads of a particular individual at a specific locus, as a read length histogram and verified by read alignment (Geneious Alignment—default settings) and manually edited [40]. Only individuals with five or more reads for a given locus were genotyped.

Read alignments of a subset of 16 individuals, with 10 or more sequence reads, from the Celtic Sea dataset (six PCR size-class-combinations) were screened for homoplasy. The aligned sequences were scrutinized for the presence of SNPs and indels that were not part of the microsatellite repeat structure and would not change the amplicon size. The SNP or indel had to occur in at least 20% of the reads to be considered as homoplasy.

Correspondence between GBS and ABI capillary-based genotyping data [32] was fitted to a binomial model [41] (electronic supplementary material, equation S1). The impact of several factors on GBS–ABI correspondence was evaluated. These factors included microsatellite type (e.g. mono-, di-), PCR size-class-combination (3-, 6- or 18-PCR), and read depth used for genotyping (broken into 2 and 3 read depth categories). Model fit was compared using the finite-sample Akaike information criterion (AIC) [42–46] (electronic supplementary material, equations S3 and S4).

Binomial proportions of the correspondence data were modelled for a restricted subset of the data, 16 individuals from the Celtic Sea (‘Celtic Sea’ dataset), for which data existed across all PCR size-class-combinations. Differences in correspondence proportions among PCR size-class-combinations were evaluated using Bonferroni-corrected *z*-tests [47]. The effectiveness of PCR size-class-combinations on the total number of reads produced for the Celtic Sea data was then evaluated. Total reads were modelled as a function of PCR size-class-combination, locus-specific primer and universal primer type, in addition to potential intrinsic confounding variables, such as inter-locus or inter-individual variability. A multinomial probability likelihood model [41] (electronic supplementary material, equation S2) was employed to evaluate model support using AIC. Bonferroni-corrected Mann–Whitney tests [47] were performed on total, median and maximum number of reads per individual between the PCR size-class-combinations to assess significant differences in the performance of PCR size-class-combinations.

## 3. Results

Sequencing of the two 1/4 regions of a PicoTiterPlate resulted in 228 246 reads for region 1 and 226 848 for region 2. Read length ranged from 54 to 1200 bp with an average read length of 275 bp (s.d. 84 bp). A total of 95.5% of reads had a quality score higher than Q20 and the average quality score was Q35.9. The proportions of A, C, G and T nucleotides were 27.0%, 22.7%, 26.7% and 23.6%, respectively, with a GC content of 49.4%.

A total of 180 054 reads were successfully assigned to a specific individual and locus combinations (electronic supplementary material, table S4). The recovered reads per individual (combined across all loci) ranged from 57 to 9192 (median: 999) (electronic supplementary material, table S3), and the number of reads per locus (combined over all individuals) ranged from 33 to 11 197 (median: 2294). The numbers of reads per genotype ranged from 0 to 1097 (median: 12). Analyses of PCR size-class-combinations resulted in 3325 successful genotypes from 5088 possible calls, with 3196 genotypes used for correspondence checks with the ABI-capillary genotyping data. Of the 53 loci analysed, 10 loci had low numbers of sequence reads preventing genotyping in more than 50% of the individuals (electronic supplementary material, table S5).

In total, 529 genotypes were screened for homoplasy, which was detected in 32% of the genotypes and was present in 38 loci. SNPs represented 80% of the homoplasy with 20% represented by indels.

AICc model selection for the Celtic Sea data demonstrated that PCR size-class-combination was the only important variable for predicting read yield. There was no support for other modelled variables (e.g. forward or reverse primers) or confounding variables (such as inter-individual and among-locus variation; table 1). Correspondence between the full GBS and ABI datasets was best explained by a model incorporating read depth and microsatellite motif type (table 2). PCR size-class-combination had an effect on the GBS–ABI correspondence in the Celtic Sea data (electronic supplementary material, table S6), with significantly higher correspondence for both 3-PCR and 6-PCR when either is compared with 18-PCR. However, there was no significant difference between 3- and 6-PCR (electronic supplementary material, table S6). In addition, significantly more reads per individual were observed for 3- and 6-PCR when compared with 18-PCR, although again, there was no significant difference between the two (electronic supplementary material, table S7). No significant differences were noted for median number of reads among size-class-combinations (electronic supplementary material, table S8). Maximum number of reads per individual showed a significant difference between 6-PCR and 18-PCR only (electronic supplementary material, table S9).

**Table 1.** AICc model selection for the reduced (‘Celtic Sea’) dataset on the read yield. (Sample size is 90194. *K* is the number of parameters estimated for a given model structure, LogL is the log-likelihood of the model, AICc is the finite-sample AIC score for the model, dAICc is the difference in AICc score between the given model and the optimal model score, and Post. Prob is the model posterior probability.)

model description	*K*	LogL	AICc	dAICc	Post. Prob.
PCR	3	−87 627	175 260	0	1.000
tails	4	−123 574	247 157	71 896	0
forward	6	−143 882	287 776	112 516	0
reverse	8	−157 688	315 391	140 131	0
PCR × tails	12	−210 436	420 896	245 635	0
individuals	16	−216 345	432 722	257 461	0
forward × tails	24	−266 358	532 764	357 504	0
PCR × reverse	24	−245 065	490 177	314 917	0
reverse × tails	32	−281 170	562 403	387 143	0
locus	53	−318 574	637 253	461 993	0
PCR × reverse × tails	96	−367 637	735 467	560 206	0

**Table 2.** Model selection using AICc on the correspondence between 454 microsatellites and ABI microsatellites, using the full dataset. (*K* is the number of parameters estimated for a given model structure, LogL is the log-likelihood of the model, AICc is the finite-sample AIC score for the model, dAICc is the difference in AICc score between the given model and the optimal model score, and Post. Prob is the model posterior probability.)

model	model comments	*K*	LogL	AICc	dAICc	Post. Prob.
no effects	average over all data	1	−1724.64	3451.28	131.96	2.22 × 10^−29^
no. reads (2)	0–5 versus 5 + reads	2	−1693.93	3391.87	72.55	1.77 × 10^−16^
no. reads (3)	0–5 versus 5–10 versus 10 + reads	3	−1676.04	3358.10	38.78	3.80 × 10^−9^
MST type		5	−1693.54	3397.11	77.79	1.29 × 10^−17^
reads (2) by MST type	0–5 versus 5 + reads	10	−1663.76	3347.59	28.27	7.26 × 10^−7^
reads (3) by MST type	0–5 versus 5–10 versus 10 + reads	15	−1644.58	3319.32	0.00	0.999
PCR (3,6) v. 18		2	−1706.09	3416.18	96.86	9.27 × 10^−22^
PCR 3,6,18		3	−1706.08	3418.16	98.84	3.45 × 10^−22^
reads (2) by PCR 3,6,18	0–5 versus 5 + reads	6	−1675.28	3362.58	43.26	4.03 × 10^−10^
reads (3) by PCR 3,6,18	0–5 versus 5–10 versus 10 + reads	9	−1658.37	3334.79	15.47	0.0004

## 4. Discussion

This study demonstrates the potential for NGS-based GBS as a method for microsatellite genotyping using *de novo* and existing capillary/gel electrophoresis-based multiplex marker panels. It also illustrates the potential for a rapid and cost-effective method for microsatellite GBS, that can take advantage of modern NGS platforms, using combinatorial barcoding for implementation in large-scale population genetics studies. This method provides access to the underlying sequence data, providing an additional advantage over traditional fragment length genotyping by resolving issues of size homoplasy and revealing potentially hidden genetic variation in the amplicons.

Analysis of the Celtic Sea data indicates that size-class combination is the controlling factor for both sequence yield and GBS–ABI correspondence. Both the 3- and 6-PCR size class conditions outperformed 18-PCR, with no significant differences between the 3- and the 6-PCR conditions observed. It is possible that 6-PCR would not have performed as well as 3-PCR in the absence of pre-optimization [32]. However, the increased number of PCR reactions and DNA quantification steps in the 18-PCR method may have introduced quantification errors that were amplified at the pooling stage, lowering read yield, and hence correspondence. As such, it is proposed that future studies minimize the number of steps to reduce variability among PCRs.

**Figure 2.**
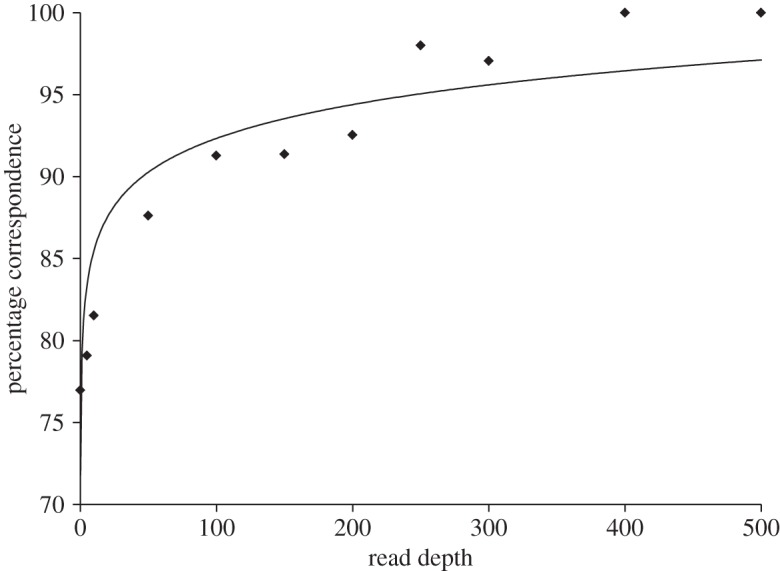
Correspondence of the GBS microsatellite data with ABI data for the full dataset. The *y*-axis represents the percentage of corresponding genotype calls of all genotype calls. The *x*-axis represents the increasing minimum threshold of read depth required for making a genotype call. The applied thresholds were 5, 10, 50, 100, 150, 200, 250, 300, 400 and 500 reads.

Binladen *et al.* [48] reported biases in sequence recovery with 454 NGS as a result of different base pair composition of barcodes. In contrast to this, the current study found no bias in sequence recovery. Read depth had a significant effect on the genotyping correspondence between the GBS and ABI-based datasets, with correspondence rapidly increasing with sequence depth (figure 2). It is unlikely that complete agreement between these two approaches is achievable, owing to inherent errors in capillary electrophoresis, genotype calls and GBS platform-specific sequencing errors. ABI-based genotype calling from electropherograms can be obscured by spectral bleeding, cross-talk between capillaries and fluctuations in instrument parameters [30]. Similarly, for 454 sequence amplicon reads, error rates have been reported as high as 6% for our study species, *G. morhua* [39]. While the error rate for 454 sequencing can be relatively high, amplicon sequencing, as used here, will be further affected by PCR-induced errors. It is therefore important to allow for some errors in barcodes and primers. When employing other NGS platforms this requirement may be alleviated by the increased read yield of these platforms.

This study represents an example of GBS using established microsatellite multiplex panels that were developed for analysis on conventional capillary/gel-based systems and contain loci with fragment sizes commonly reported in microsatellite studies (approx. 100–500 bp) [28,32,49,50]. Owing to the large size range of alleles in the established microsatellite multiplex panel, the choice of NGS platform was restricted to 454, as it produces up to 1 million reads of up to 700 bp [51]. While the 454 NGS platform has the capacity to produce long sequence reads, the sequence yield is significantly lower than in other platforms: for example, the Ion Torrent platform ([52]; Life Technologies) produces up to six million 400 bp reads on their 318^®^ chip [53] and the Illumina^®^ Miseq platform [54]; Illumina Inc. San Diego, CA, USA) currently produces 20–30 million paired end reads of 2×300 bp. The short read length of these platforms limits application to relatively short amplicons (including barcode, primers and repetitive sequence). However, this limitation may be mitigated by *de novo* development of microsatellite markers, the use of partial existing microsatellite panels (i.e. loci with amplicon sizes within the read length limitation of the chosen NGS platform), or the redesign of primers for existing microsatellite markers to produce shorter amplicons.

The current study was not optimized for population genetics scale genotyping as a larger number of loci and a lower number of individuals were analysed than is often used in population genetic studies (cf. [32]). However, in an effort to address multiple questions about the feasibility of GBS of microsatellites, it was judged advantageous to include a larger number of loci than needed for many population genetic studies. In addition, if this method was used on *de novo* developed loci, the success rate from microsatellite containing sequences to genotyped loci is only known *post hoc* and hence a larger number of initial loci would increase the likelihood of generating a sufficient number of informative loci. For deployment of the approach described here, in a population genetics setting, it may be more beneficial to interrogate fewer loci, but more individuals. This could be facilitated by increasing the number of forward and reverse combinatorial barcodes used to tag an increased number of individuals.

Manual scoring of conventional capillary and gel-based electrophoresis fragment length polymorphism is time consuming, and therefore carries a significant financial cost to genetic studies. This study describes an approach that does not require manual correction of internal size standards or genotype calls owing to spectral bleed-through, thus reducing the genotyping time. In addition, the availability of the underlying sequence data lends itself to the development of automated genotyping [55]. The numbers of loci that can be multiplexed in capillary or gel-based electrophoresis in conventional studies are limited by the availability of fluorescent labels/detection channels and fragment size overlaps (rarely more than 12 loci per multiplex) [56]. The GBS approach described in this study using PCR-incorporated combinatorial barcoding has no limitations on the number of markers (other than number of sequences produced by the chosen NGS platform), as size overlap does not affect sequence yield.

The high proportion of homoplasy (32%) observed is in concordance with other studies addressing the prevalence of homoplasy [57–60]. This homoplasy would have been undetectable using traditional ABI-based microsatellite genotyping as the amplicon size would be unaffected. Previous detection methods include single-strand conformation polymorphism analysis and direct sequencing, with or without cloning, however, these detection methods can be laborious [57–60]. It should be noted that low read depth may prevent distinguishing homoplasy from true mutations and sequencing/PCR error. However, the purpose of the analysis in the current study was to explore the capacity to detect and quantify prevalence of homoplasy using GBS-based methods. The prevalence of homoplasy implies that allelic diversity in fragment size-based studies is probably substantially underestimated. Consequently, inferred population structures in these studies may also underestimate true levels of genetic variability. Aside from homoplasy, determining microsatellite repeat numbers could improve genetic diversity comparisons [61]. Essentially, accessing the molecular structure of the microsatellite markers will increase our understanding of the mutation model of the studied loci and thus improve the quality of the information retrieved from the data. In addition, access to actual sequence lengths and sequence information will greatly facilitate inter-laboratory calibration and data storage in repositories as sequence files offering a significant advantage.

This study presents a novel method for microsatellite GBS using individual combinatorial barcoding that can be faster and cheaper than current approaches while offering better and more data.

## Supplementary Material

Supplementary_tables_Royal_open_sci.docx

## Data accessibility

The genotype datasets supporting this article are provided as part of the electronic supplementary material, table S10). All the scripts used are available on github (https://github.com/egenomics/micomba).
