# Supplementary material for: A novel method of microsatellite genotyping-by-sequencing using individual combinatorial barcoding
Source: R Soc Open Sci. 2016 Jan 20;3(1):150565. doi: 10.1098/rsos.150565 (PMC4736940; doi:10.1098/rsos.150565)
Supplement: Supplementary_tables_Royal_open_sci.docx [file rsos150565supp1.docx]

**ESM: Tables 1-9 and Equations**

| **ESM: Table 1** Multiplex panels used in the three experimental conditions with the size ranges of markers. | | | | | | | | | | |
| --- | --- | --- | --- | --- | --- | --- | --- | --- | --- | --- |
| **Size-class-combination 1** | |  | **Size-class-combination 2** | | | |  | **Size-class-combination 3** | | |
| **3 PCR** | |  | **6 PCR** | | | |  | **18 PCR** | | |
|  |  |  |  | |  | |  |  |  | |
| **Multiplex 1** | **Size-class I** |  | **Multiplex 1** | | **Size-class mixed** | |  | **Multiplex 1a** | **Size-class I** | |
| A08_T3 | 163–187 |  | A08_T3 | | 163–187 | |  | A08_T3 | 163–187 | |
| A16_M13 | 156–194 |  | A16_M13 | | 156–194 | |  | A16_M13 | 156–194 | |
| C15_Hill | 169–205 |  | C15_Hill | | 169–205 | |  | C15_Hill | 169–205 | |
| B03_T3 | 147–161 |  | B19_T3 | | 242–320 | |  |  |  | |
| B30_Neo | 151–169 |  | C28_Neo | | 255–282 | |  | **Multiplex 1b** | **Size-class II** | |
| C01_M13 | 115–195 |  | C40_M13 | | 276–277 | |  | B19_T3 | 242–320 | |
| C14_Hill | 146–166 |  | D14_Hill | | 228–276 | |  | C28_Neo | 255–282 | |
| B01_Hill | 129–213 |  | A43_T3 | | 309–369 | |  | C40_M13 | 276–277 | |
| B15_T3 | 170–178 |  | B38_Neo | | 388–415 | |  | D14_Hill | 228–276 | |
| C13_Neo | 158–176 |  | C36_Hill | | 319–352 | |  |  |  | |
| C17_M13 | 154–190 |  | D30_M13 | | 317–333 | |  | **Multiplex 1c** | **Size-class III** | |
| A11_Hill | 163–175 |  |  | |  | |  | A43_T3 | 309–369 | |
| B33_M13 | 120–135 |  | **Multiplex 2** | | **Size-class mixed** | |  | B38_Neo | 388–415 | |
| C08_T3 | 145–185 |  | B03_T3 | | 147–161 | |  | C36_Hill | 319–352 | |
| D46_Neo | 139–155 |  | B30_Neo | | 151–169 | |  | D30_M13 | 317–333 | |
| A04_Neo | 143–211 |  | C01_M13 | | 115–195 | |  |  |  | |
| B07_M13 | 163–187 |  | C14_Hill | | 146–166 | |  | **Multiplex 2a** | **Size-class I** | |
| A03_Hill | 158–170 |  | A18_M13 | | 203–243 | |  | B03_T3 | 147–161 | |
| A20_Neo | 164–182 |  | A19_T3 | | 242–274 | |  | B30_Neo | 151–169 | |
|  |  |  | C20_Neo | | 227–239 | |  | C01_M13 | 115–195 | |
| **Multiplex 2** | **Size-class II** |  | D12_Hill | | 251–291 | |  | C14_Hill | 146–166 | |
| B19_T3 | 242–320 |  | A34_M13 | | 297–321 | |  |  |  | |
| C28_Neo | 255–282 |  | A37_Neo | | 265–489 | |  | **Multiplex 2b** | **Size-class II** | |
| C40_M13 | 276–277 |  | B12_T3 | | 346–398 | |  | A18_M13 | 203–243 | |
| D14_Hill | 228–276 |  |  | |  | |  | A19_T3 | 242–274 | |
| A18_M13 | 203–243 |  | **Multiplex 3** | | **Size-class mixed** | |  | C20_Neo | 227–239 | |
| A19_T3 | 242–274 |  | B01_Hill | | 129–213 | |  | D12_Hill | 251–291 | |
| C20_Neo | 227–239 |  | B15_T3 | | 170–178 | |  |  |  | |
| D12_Hill | 251–291 |  | C13_Neo | | 158–176 | |  | **Multiplex 2c** | **Size-class III** | |
| A33_M13 | 233–297 |  | C17_M13 | | 154–190 | |  | A34_M13 | 297–321 | |
| B29_Neo | 262–281 |  | A33_M13 | | 233–297 | |  | A37_Neo | 265–489 | |
| C22_Hill | 225–273 |  | B29_Neo | | 262–281 | |  | B12_T3 | 346–398 | |
| A22_Neo | 214–243 |  | C22_Hill | | 225–273 | |  |  |  | |
| D15_Hill | 244–256 |  | A39_Hill | | 356–416 | |  | **Multiplex 3a** | **Size-class I** | |
| D43_T3 | 260–292 |  | C30_Neo | | 337–343 | |  | B01_Hill | 129–213 | |
| B28_Neo | 226–238 |  | C42_M13 | | 345–358 | |  | B15_T3 | 170–178 | |
| A30_T3 | 245–269 |  | D37_T3 | | 313–333 | |  | C13_Neo | 158–176 | |
| C35_M13 | 233–348 |  |  | |  | |  | C17_M13 | 154–190 | |
| D05_Hill | 230–270 |  | **Multiplex 4** | | **Size-class mixed** | |  |  |  | |
|  |  |  | A11_Hill | | 163–175 | |  | **Multiplex 3b** | **Size-class II** | |
| **Multiplex 3** | **Size-class III** |  | B33_M13 | | 120–135 | |  | A33_M13 | 233–297 | |
| A43_T3 | 309–369 |  | C08_T3 | | 145–185 | |  | B29_Neo | 262–281 | |
| B38_Neo | 388–415 |  | D46_Neo | | 139–155 | |  | C22_Hill | 225–273 | |
| C36_Hill | 319–352 |  | A22_Neo | | 214–243 | |  |  |  | |
| D30_M13 | 317–333 |  | D15_Hill | | 244–256 | |  | **Multiplex 3c** | **Size-class III** | |
| A34_M13 | 297–321 |  | A31_T3 | | 314–374 | |  | A39_Hill | 356–416 | |
| A37_Neo | 265–489 |  | D21_Neo | | 337–389 | |  | C30_Neo | 337–343 | |
| B12_T3 | 346–398 |  |  | |  | |  | C42_M13 | 345–358 | |
| A39_Hill | 356–416 |  | **Multiplex 5** | | **Size-class mixed** | |  | D37_T3 | 313–333 | |
| C30_Neo | 337–343 |  | A04_Neo | | 143–211 | |  |  |  | |
| C42_M13 | 345–358 |  | B07_M13 | | 163–187 | |  | **Multiplex 4a** | **Size-class I** | |
| D37_T3 | 313–333 |  | D43_T3 | | 260–292 | |  | A11_Hill | 163–175 | |
| A31_T3 | 314–374 |  | B28_Neo | | 226–238 | |  | B33_M13 | 120–135 | |
| D21_Neo | 337–389 |  | D10_Neo | | 308–312 | |  | C08_T3 | 145–185 | |
| D10_Neo | 308–312 |  | D35_M13 | | 316–360 | |  | D46_Neo | 139–155 | |
| D35_M13 | 316–360 |  |  | |  | |  |  |  | |
| B36_Hill | 384–396 |  | **Multiplex 6** | | **Size-class mixed** | |  | **Multiplex 4b** | **Size-class II** | |
| C31_Neo | 332–341 |  | A03_Hill | | 158–170 | |  | A22_Neo | 214–243 | |
|  |  |  | A20_Neo | | 164–182 | |  | D15_Hill | 244–256 | |
|  |  |  | A30_T3 | | 245–269 | |  |  |  | |
|  |  |  | C35_M13 | | 233–348 | |  | **Multiplex 4c** | **Size-class III** | |
|  |  |  | D05_Hill | | 230–270 | |  | A31_T3 | 314–374 | |
|  |  |  | B36_Hill | | 384–396 | |  | D21_Neo | 337–389 | |
|  |  |  | C31_Neo | | 332–341 | |  |  |  | |
|  |  |  |  | |  | |  | **Multiplex 5a** | **Size-class I** | |
|  |  |  |  | |  | |  | A04_Neo | 143–211 | |
|  |  |  |  | |  | |  | B07_M13 | 163–187 | |
|  |  |  |  | |  | |  |  |  | |
|  |  |  |  | |  | |  | **Multiplex 5b** | **Size-class II** | |
|  |  |  |  | |  | |  | D43_T3 | 260–292 | |
|  |  |  |  | |  | |  | B28_Neo | 226–238 | |
|  |  |  |  | |  | |  |  |  | |
|  |  |  |  | |  | |  | **Multiplex 5c** | **Size-class III** | |
|  |  |  |  | |  | |  | D10_Neo | 308–312 | |
|  |  |  |  | |  | |  | D35_M13 | 316–360 | |
|  |  |  |  | |  | |  |  |  | |
|  |  |  |  | |  | |  | **Multiplex 6a** | **Size-class I** | |
|  |  |  |  | |  | |  | A03_Hill | 158–170 | |
|  |  |  |  | |  | |  | A20_Neo | 164–182 | |
|  |  |  |  | |  | |  |  |  | |
|  |  |  |  | |  | |  | **Multiplex 6b** | **Size-class II** | |
|  |  |  |  | |  | |  | A30_T3 | 245–269 | |
|  |  |  |  | |  | |  | C35_M13 | 233–348 | |
|  |  |  |  | |  | |  | D05_Hill | 230–270 | |
|  |  |  |  | |  | |  |  |  | |
|  |  |  |  | |  | |  | **Multiplex 6c** | **Size-class III** | |
|  |  |  |  | |  | |  | B36_Hill | 384–396 | |
|  |  |  |  | |  | |  | C31_Neo | 332–341 | |
| **ESM: Table 2** Barcode and universal primer sequences | | | | | | | | | | |
| Oligonucleotide type | | | | | Name | | Sequence 5' → 3' | | | |
|  | | | | |  | |  | | | |
| Forward universal primer | | | | | T3 | | AATTAACCCTCACTAAAGGG | | | |
| Forward universal primer | | | | | M13 Reverse | | GGATAACAATTTCACACAGG | | | |
| Forward universal primer | | | | | Hill | | TGACCGGCAGCAAAATTG | | | |
| Forward universal primer | | | | | Neomycin rev | | AGGTGAGATGACAGGAGATC | | | |
|  | | | | |  | |  | | | |
| Reverse universal primer | | | | | CAG | | CAGTCGGGCGTCATCA | | | |
|  | | | | |  | |  | | | |
| Barcode_F1 | | | | | MID-1 | | ACGAGTGCGT | | | |
| Barcode_F2 | | | | | MID-2 | | ACGCTCGACA | | | |
| Barcode_F3 | | | | | MID-3 | | AGACGCACTC | | | |
| Barcode_F4 | | | | | MID-4 | | AGCACTGTAG | | | |
| Barcode_F5 | | | | | MID-5 | | ATCAGACACG | | | |
| Barcode_F6 | | | | | MID-6 | | ATATCGCGAG | | | |
| Barcode_F7 | | | | | MID-7 | | CGTGTCTCTA | | | |
| Barcode_F8 | | | | | MID-10 | | TCTCTATGCG | | | |
| Barcode_F9 | | | | | MID-11 | | TGATACGTCT | | | |
| Barcode_F10 | | | | | MID-13 | | CATAGTAGTG | | | |
| Barcode_F11 | | | | | MID-14 | | CGAGAGATAC | | | |
| Barcode_F12 | | | | | MID-15 | | ATACGACGTA | | | |
|  | | | | |  | |  | | | |
| Barcode_R1 | | | | | MID-16 | | TCACGTACTA | | | |
| Barcode_R2 | | | | | MID-17 | | CGTCTAGTAC | | | |
| Barcode_R3 | | | | | MID-19 | | TGTACTACTC | | | |
| Barcode_R4 | | | | | MID-21 | | CGTAGACTAG | | | |
| Barcode_R5 | | | | | MID-22 | | TACGAGTATG | | | |
| Barcode_R6 | | | | | MID-23 | | TACTCTCGTG | | | |
| Barcode_R7 | | | | | MID-24 | | TAGAGACGAG | | | |
| Barcode_R8 | | | | | MID-27 | | ACGCGAGTAT | | | |

| **ESM: Table 3** The barcode combinations used to identify individual amplicons. Reads recovered per individual after read sorting are shown under the individual code. The total number of recovered reads was 180 054. CS = Celtic Sea; WI = West of Ireland; 3-PCR is size-class combination 1; 6-PCR is size-class combination 2; and 18-PCR is size-class combination 3. | | | | | | | | | | |
| --- | --- | --- | --- | --- | --- | --- | --- | --- | --- | --- |
|  | **Barcode** | **Barcode** | **Barcode** | **Barcode** | **Barcode** | **Barcode** | **Barcode** | **Barcode** |  |  |
|  | **_R1** | **_R2** | **_R3** | **_R4** | **_R5** | **_R6** | **_R7** | **_R8** |  |  |
| **Barcode** | **CS_09** | **CS_10** | **CS_11** | **CS_12** | **CS_13** | **CS_14** | **CS_15** | **CS_16** |  | **3** |
| **_F12** | **772** | **4236** | **1022** | **4366** | **415** | **1116** | **277** | **4381** |  | **PCR** |
| **Barcode** | **CS_01** | **CS_02** | **CS_03** | **CS_04** | **CS_05** | **CS_06** | **CS_07** | **CS_08** |  |  |
| **_F11** | **499** | **3424** | **648** | **2562** | **328** | **764** | **216** | **2993** |  |  |
| **Barcode** | **CS_09** | **CS_10** | **CS_11** | **CS_12** | **CS_13** | **CS_14** | **CS_15** | **CS_16** |  | **6** |
| **_F10** | **1695** | **9192** | **3010** | **8988** | **1261** | **2690** | **667** | **8656** |  | **PCR** |
| **Barcode** | **CS_01** | **CS_02** | **CS_03** | **CS_04** | **CS_05** | **CS_06** | **CS_07** | **CS_08** |  |  |
| **_F9** | **511** | **3627** | **921** | **3169** | **462** | **1038** | **234** | **3158** |  |  |
| **Barcode** | **WI_25** | **WI_26** | **WI_27** | **WI_28** | **WI_29** | **WI_30** | **WI_31** | **WI_32** |  |  |
| **_F8** | **136** | **762** | **285** | **779** | **90** | **181** | **57** | **605** |  |  |
| **Barcode** | **WI_17** | **WI_18** | **WI_19** | **WI_20** | **WI_21** | **WI_22** | **WI_23** | **WI_24** |  |  |
| **_F7** | **475** | **2637** | **988** | **2370** | **371** | **804** | **179** | **2181** |  |  |
| **Barcode** | **WI_09** | **WI_10** | **WI_11** | **WI_12** | **WI_13** | **WI_14** | **WI_15** | **WI_16** |  |  |
| **_F6** | **876** | **5108** | **1907** | **4970** | **641** | **1361** | **302** | **3539** |  | **18** |
| **Barcode** | **WI_01** | **WI_02** | **WI_03** | **WI_04** | **WI_05** | **WI_06** | **WI_07** | **WI_08** |  | **PCR** |
| **_F5** | **900** | **6054** | **1978** | **5518** | **704** | **1559** | **339** | **4204** |  |  |
| **Barcode** | **CS_25** | **CS_26** | **CS_27** | **CS_28** | **CS_29** | **CS_30** | **CS_31** | **CS_32** |  |  |
| **_F4** | **826** | **4996** | **1646** | **4719** | **605** | **1332** | **284** | **3626** |  |  |
| **Barcode** | **CS_17** | **CS_18** | **CS_19** | **CS_20** | **CS_21** | **CS_22** | **CS_23** | **CS_24** |  |  |
| **_F3** | **891** | **5163** | **1724** | **5028** | **622** | **1515** | **283** | **3740** |  |  |
| **Barcode** | **CS_09** | **CS_10** | **CS_11** | **CS_12** | **CS_13** | **CS_14** | **CS_15** | **CS_16** |  |  |
| **_F2** | **279** | **1478** | **472** | **1476** | **184** | **367** | **92** | **1009** |  |  |
| **Barcode** | **CS_01** | **CS_02** | **CS_03** | **CS_04** | **CS_05** | **CS_06** | **CS_07** | **CS_08** |  |  |
| **_F1** | **384** | **2179** | **716** | **1996** | **195** | **547** | **114** | **1408** |  |  |

| **ESM: Table 4** Number of barcodes and locus-specific primers identified in the reads. | |
| --- | --- |
| Identified primers and barcodes | No. of reads |
| Two barcodes and two matching primers | 180054 |
| Two barcodes and two non-matching primers | 2175 |
| Two barcodes and one primer | 4843 |
| Two barcodes and no primers | 801 |
| One barcode | 205593 |
| No barcodes | 61628 |
| total | **455094** |

| **ESM: Table 5** Loci with less than 50% of individuals genotyped. | |
| --- | --- |
| Loci name | Percentage of individuals genotyped |
| A03_Hill | 1.0% |
| C35_M13 | 7.3% |
| D10_Neo | 14.6% |
| A30_T3 | 25.0% |
| B19_T3 | 39.6% |
| B12_T3 | 41.7% |
| D37_T3 | 41.7% |
| A19_T3 | 42.7% |
| C31_Neo | 45.8% |
| B36_Hill | 46.9% |

| **ESM: Table 6** Bonferroni corrected 2-tail p-values for z-tests for the proportion of correspondence between 454 and ABI performed on the reduced (“Celtic Sea”) data set. | | | | |
| --- | --- | --- | --- | --- |
|  | 3 PCR | 6 PCR | 18 PCR | Proportion |
| 3 PCR |  |  |  | 0.8298 |
| 6 PCR | 1.0000 |  |  | 0.8333 |
| 18 PCR | 0.0006 | 0.0005 |  | 0.7512 |

| **ESM: Table 7** Bonferroni corrected 2-tail p-values Mann-Whitney tests performed on the reduced (“Celtic Sea”) data set for differences in the total number of reads per individual by PCR condition type. | | | | |
| --- | --- | --- | --- | --- |
|  | 18 PCR | 6 PCR | 3 PCR | Average total reads per individual |
| 18 PCR |  |  |  | 806 |
| 6 PCR | 0.0002 |  |  | 3080 |
| 3 PCR | 0.0406 | 0.0907 |  | 1751 |

| **ESM: Table 8** Bonferroni corrected 2-tail p-values Mann-Whitney tests performed on the reduced (“Celtic Sea”) data set for differences in the median number of reads per individual by PCR condition type. | | | | |
| --- | --- | --- | --- | --- |
|  | 18 PCR | 6 PCR | 3 PCR | Average median reads per individual |
| 18 PCR |  |  |  | 11 |
| 6 PCR | 1.0000 |  |  | 20 |
| 3 PCR | 0.5590 | 1.0000 |  | 19 |

| **ESM: Table 9** Bonferroni corrected 2-tail p-values Mann-Whitney tests performed on the reduced (“Celtic Sea”) data set for differences in the maximum number of reads per individual by PCR condition type. | | | | |
| --- | --- | --- | --- | --- |
|  | 18 PCR | 6 PCR | 3 PCR | Average maximum reads per individual |
| 18 PCR |  |  |  | 50 |
| 6 PCR | 0.0168 |  |  | 402 |
| 3 PCR | 0.2390 | 0.6619 |  | 165 |

**ESM: Equations**

We modelled the likelihood fit of the proportional correspondence between 454 GBS and ABI genotyping using a binomial model. The sufficient statistic for the log-likelihood of a binomial is given by:

Eq 1. $LogL\propto a\text{*}Ln[p]+(n-a)\text{*}Ln[1-p]$(Edwards 1992),

where ***p*** is the binomial rate parameter or proportional correspondence, ***a*** is the number of correct correspondences between 454 GBS and ABI capillary-based genotyping data, and ***n*** is the total number of potential correspondences. In the present study, ***n*** = 3196.

For a restricted subset of the data, 16 individuals from the Celtic Sea (“Celtic Sea” data set), for which overlapping data existed across all PCR size-class-combinations, we modelled total reads as a function of PCR size-class-combination, locus-specific primer, and universal primer type, in addition to potential intrinsic confounding intrinsic variables, such as inter-locus or inter-individual variability. We employed a multinomial probability likelihood model:

Eq. 2: $LogL\propto\sum_{1}^{R} a_{R}\text{*}Ln\left( p_{R} \right)$ (Edwards 1992).

In equation 2, ***R*** is the number of rate classes in the proposed model structure, ***a*** is the observed number of reads in that class, and ***p*** = a/n, where n is total reads in the Celtic Sea data (n = 90194), that is, the observed proportion of reads in that class. Read data were modelled to compare the effectiveness of the different variables, as well as potential interactive effects between the variables, on total read count. As with the correspondence data, we evaluated model fit using ***AICc***.

In all cases, the simplest model would be a “no effects” model, with a single binomial rate characterizing all of the data. More complex models would then propose different values of ***p*** based on different variable factors, for example a model could potentially estimate a separate value of ***p*** for each of the three PCR size-classes. More complex models will tend to have better fit to the data, as they can be “tuned” to explain specific variations in the proportional correspondence. However, they do this at the expense of increase parameter estimation, which can lead to large variance in parameter estimates (Burnham and Anderson 2002, 2004). To select among models we employed the finite-sample Akaike Information Criterion (AICc) (Akaike 1973, 1974; Hurvich and Tsai 1989), which measures model fit to the observed data, penalized for increased model; complexity.

Eq. 3: $AICc=-2LogL+2K+\frac{2K(K+1)}{n-K-1}$ (Burnham and Anderson 2002).

In equation 3, ***LogL*** is the log-likelihood fit of the model to the data, ***K*** is the number of estimated parameters for the model, and ***n*** is the sample size. ***AICc*** is proportional to the parameter-corrected model likelihood by:

Eq. 4. $ModelL\propto e^{\frac{-1}{2}\Delta AICc}$(Burnham and Anderson 2002),

where ***ΔAICc*** is the difference in ***AICc*** score between any given model and the optimal model score. This can then be converted into a model posterior probability for the set of models under consideration by dividing each model’s likelihood by the sum over all models’ likelihoods.

References

Akaike, H. 1973. Information theory as an extension of the maximum likelihood principle. Pp. 267-281. *In* B. N. Petrov, and F. Csaki, eds. Second International Symposium on Information Theory. Akademiai Kiado, Budapest.

Akaike, H. 1974. A new look at the statistical model identification. Institute of Electrical and Electronics Engineers Transactions on Automatic Control AC-19:716-723.

Burnham, K. P., and D. R. Anderson. 2002. Model Selection and Multimodel Inference: A Practical Information-Theoretic Approach. Springer, New York.

Burnham, K. P., and D. R. Anderson. 2004. Multimodel inference: Understanding AIC and BIC in model selection. Sociological Methods and Research 33(2):261-304.

Hurvich, C. M., and C.-L. Tsai. 1989. Regression and time series model selection in small samples. Biometrika 76(2):297-307.
